# Association of *NKAPL* rs1635 With Cognitive Function in Early-Onset Schizophrenia

**DOI:** 10.3389/fgene.2022.941171

**Published:** 2022-06-21

**Authors:** Yang Yang, Yi Su, Guiming Wei, Zhewei Kang, Zhe Lu, Yundan Liao, Tianlan Lu, Hao Yan, Weihua Yue, Ying Qin, Yuyanan Zhang

**Affiliations:** ^1^ Peking University Sixth Hospital, Peking University Institute of Mental Health, NHC Key Laboratory of Mental Health (Peking University), National Clinical Research Center for Mental Disorders (Peking University Sixth Hospital), Beijing, China; ^2^ Peking University HuiLongGuan Clinical Medical School, Beijing HuiLongGuan Hospital, Beijing, China; ^3^ Department of Neurology, Shandong Daizhuang Hospital, Jining, China; ^4^ PKU-IDG/McGovern Institute for Brain Research, Peking University, Beijing, China; ^5^ Chinese Institute for Brain Research, Beijing, China; ^6^ The Second People’s Hospital of Guizhou Province, Guiyang, China

**Keywords:** NKAPL, rs1635, early-onset schizophrenia, cognitive function, neuron migration

## Abstract

**BACKGROUND:** Schizophrenia is a severe mental disorder with high heritability, and cognitive dysfunction is one of the core features. Growing evidence suggests the genetic risk of schizophrenia may contribute to cognitive impairments. The variant rs1635 (nucleotide sequence: c.455C>A; amino acid sequence: T152N) located on the (NFKB activating protein like) *NKAPL* gene confers risk for schizophrenia and might play a role in the neurodevelopmental process, which is particularly relevant to cognitive function. However, the relationship between rs1635 and cognitive function remains unclear.

**METHODS:** A total of 130 patients with early-onset schizophrenia (EOS) and 300 patients with adult-onset schizophrenia (AOS) of Han Chinese were recruited and underwent neurocognitive tests by using the MATRICS Consensus Cognitive Battery (MCCB). The *NKAPL* rs1635 was genotyped by using DNA sequencing. The peripheral blood NKAPL mRNA expression level was examined in 152T or 152N carriers (*n* = 20) in EOS patients, by using the qRT-PCR. The phosphorylation level of NAKPL T152N polymorphism was detected by cell experiments. In utero electroporation of mouse embryos was examined to explore the effect of Nkapl on neuronal migration.

**RESULTS:** Compared with rs1635 AA and AC carriers, CC (the CC genotype encodes the protein NKAPL-152T) carriers of EOS patients performed better in cognitive domain of speed of processing (*t* = 2.644, *p* = 0.009), trail making test (*t* = 2.221, *p* = 0.028) and category fluency (*t* = 2.578, *p* = 0.011). However, patients with AOS exhibited no significant differences in seven domains among the three genotype groups. There were no significant differences in cognitive performance between EOS and AOS. In EOS patients, *NKAPL* mRNA level in NKAPL-152N carriers is significantly lower than that of NKAPL-152T carriers. The phosphorylation level of NKAPL-152N is significantly decreased compared to NKAPL-152T. In utero electroporation showed that *Nkapl* deletion impairs the embryonic radial migration process.

**CONCLUSION:** The present study found that *NKAPL* rs1635 was associated with cognitive impairments and peripheral blood mRNA expression level in EOS patients. The NKAPL full-length protein is required for embryonic cortical neuronal migration. The phosphorylation level of NKAPL-152N is significantly decreased. The NKAPL T152N may affect the NAKPL mRNA expression level and embryonic cortical neuronal migration by regulating the NAKPL protein phosphorylation. These data suggest that *NKAPL* rs1635 affects cognitive function by regulating early brain development in early-onset schizophrenia.

## Introduction

Schizophrenia is a severe mental disorder with high heritable factors (McCutcheon et al., 2020). The core features of schizophrenia include positive symptoms, negative symptoms, and cognitive deficits. The cognitive deficits begin to appear in the premorbid phase (Woodberry et al., 2008), worsen in the prodromal phase (Mesholam-Gately et al., 2009), and have been fully developed at the first episode of psychosis (Seidman et al., 2010). Further, cognitive impairments are important factors associated with functional outcomes in schizophrenia (Mohamed et al., 2008). Accumulating evidence has suggested the contribution of genetic factors to cognitive deficits in schizophrenia (Zai et al., 2017), including significant genetic overlap between neurocognition and schizophrenia (Lencz et al., 2014; Ohi et al., 2018). However, there has been no effective strategy for the treatment of cognitive deficits in schizophrenia. Therefore, further understanding of the molecular mechanisms underlying cognitive deficits of schizophrenia is needed, especially the effect of genetic variants.

Early-onset schizophrenia (EOS) is defined as the first onset of schizophrenia before age 18 (Cannon et al., 1999), which is related to greater genetic and developmental factors (Rapoport et al., 2005). Neurodevelopmental processes are strongly linked to cognitive function in schizophrenia. Much evidence indicates that the pathogenesis of schizophrenia begins early in neurodevelopment including in utero adversity and obstetric complications (Jaaro-Peled and Sawa, 2020). The development of the executive function, memory, and attention is important in late childhood and adolescence (Waber et al., 2012). Patients with early-onset schizophrenia have increased disease severity compared with those with adult-onset schizophrenia, which might be related to their different developmental trajectories. The pathogenesis of EOS may be more influenced by genetics and neurodevelopment. In this background, the research on cognitive dysfunction in patients with EOS gives us some inspiration to explore how biological mechanisms affect cognitive dysfunction in patients and cognitive development in adolescents.

In recent years, genome-wide association studies (GWAS) reveal multiple common variants significantly associated with schizophrenia. (Schizophrenia Working Group of the Psychiatric Genomics Consortium, 2014). Accumulating evidence suggests the genetic risk of schizophrenia may play a role in cognitive impairments (Zai et al., 2017). Polygenic risk scores based on genome-wide association studies (GWAS) data of schizophrenia are associated with decreased cognitive abilities in nonclinical cohorts (Lencz et al., 2014; Hubbard et al., 2016). The single nucleotide polymorphism (SNP) rs1635 was associated with the risk of schizophrenia in a Chinese Han population (Yue et al., 2011; Chen et al., 2014). The rs1635 is a non-synonymous SNP located in exon one of the NFKB activating protein-like (*NKAPL*) gene resulting in a T152N substitution in the encoded protein. However, it is unclear whether *NKAPL* is related to cognitive impairments.

Therefore, we conducted this study to further explore the association between *NKAPL* variant rs1635 and cognitive function across different domains in patients with schizophrenia. We evaluated the possible role of Nkapl in the migration of neurons by using in utero electroporation and detected the phosphorylation level of the NKAPL T152N variant.

## Materials and methods

### Participants

We recruited 515 healthy individuals and a total of 430 patients with schizophrenia who are all Han Chinese, including 130 EOS and 300 AOS. The experienced psychiatrists made a diagnosis using the Structured Clinical Interview for Diagnostic and the Statistical Manual of Mental Disorders, Fourth Edition, Text Revision (DSM-IV-TR) Axis I Disorders (SCID, patient edition). Patients with any other neurological disorder, a history of severe medical illness, substance dependence, pregnancy, or treatment with electroconvulsive therapy within the past 6 months, and those with a diagnosis of any other Axis I disorder, were excluded. All healthy participants were of Chinese Han ancestry, 18- to 45-year-old and right-handed with educational attainment ≥9 years. They were assessed by psychiatrists using the SCID to exclude the presence of any psychiatric disorder. Written informed consent was given by all the participants. The study was approved by the research ethics committees of Peking University Sixth Hospital and The Second People’s Hospital of Guizhou Province.

### Assessment of Symptomatology and Cognitive Function

The severity of symptoms of patients was evaluated by trained and experienced psychiatrists using the Positive and Negative Syndrome Scale (PANSS). Cognitive function was assessed using the Chinese version (Shi et al., 2015) of the MATRICS Consensus Cognitive Battery (MCCB) (Nuechterlein et al., 2008). The MCCB includes nine tests that measure seven cognitive domains: speed of processing, attention/vigilance, working memory, verbal learning, visual learning; reasoning and problem solving, and social cognition. There are three tests included in the domain of speed of processing, that is, Trail Making Test: Part A (TMT); Category Fluency: Animal Naming Test (Fluency); Brief Assessment of Cognition in Schizophrenia: Symbol Coding Test (BACS_SC). Raw MCCB scores were standardized to T scores (mean = 50, SD = 10). Generalized linear regression was used to calculate a residual value for each MCCB score with the effect of age, sex, and educational attainment regressed out to control the confounding effect.

### Genotyping

Peripheral blood samples were collected from all subjects using EDTA tubes. Genomic DNA was extracted from whole blood with the QIAamp DNA Mini Kit according to the manufacturer’s instructions (QIAGEN, Hilden, Germany). The *NKAPL* rs1635 polymorphism was genotyped by the direct DNA sequencing method. The information of rs1635 was selected from dbSNP (http://www.ncbi.nlm.nih.gov/SNP/) and the primer sequences were as follows: 5′ GCTACCATCGTCACT 3′ and 5′ TTTCACCTCTTCGTGGGA 3’. After PCR amplification, the PCR products were purified using BigDye Terminator Cycle Sequencing Ready Reaction Kit with Ampli-Taq DNA polymerase (PE Biosystem) and were sequenced by DNA sequencing with inner primers for the cycle-sequencing reaction, and fragments were separated by electrophoresis on ABI PRISM Genetic Analyzer (Applied Biosystems, Foster City, CA, United States). All genotyping was done blind to the knowledge of the subjects’ clinical data.

### Cell Culture

Human embryonic kidney (HEK) 293T cells were maintained in Dulbecco’s Modified Eagle Medium (DMEM) containing 10% fetal bovine serum (FBS), and the expression vector [plasmid containing cytomegalovirus-enhancer chicken beta-actin promotor (pcAGGS)-NKAPL (152T)-hemagglutinin (HA)-intrinsic ribosomal entry site (IRES)-EGFP, pcAGGS-NKAPL (152N)- HA-IRES-EGFP] was transfected alone with lipofectamine 2000 reagent (Invitrogen, 11668) according to manufacturer’s instructions. After 6 h, the medium was changed to fresh DMEM containing 10% FBS and cultured for 48 h.

### Quantitative Real-Time PCR (qRT-PCR)

Total RNA was extracted from the peripheral blood mononuclear cell (PBMC) using Trizol reagent (Invitrogen). The concentration and quality of the RNA were checked by spectrophotometry and gel electrophoresis. Totally 1 μg RNA was used for reverse transcribed by using the QuantiTect Reverse Transcription Kit (Qiagen). Quantitative real-time PCR (qRT-PCR) was performed using Power SYBR Green PCR Master Mix with ABI 7500 Fast RT-PCR system (Applied Biosystems). The PCR cycling conditions included an initial denaturation step of 95°C for 10 min, 40 cycles at 95°C for 15 s, and 60°C for 1 min. The final primer concentration was 125 nM. The comparative CT method (2-∆∆CT) was used to calculate the relative level of the mRNA normalized to the beta-actin gene. All samples were measured in triplicates. Sequences of forward and reverse primers for NKAPL were 5′-ATGTTCCTCTTGGGATGGC-3’and 5′-AGT​TGC​GGA​ATC​TTG​GGA​G-3′, respectively. Beta-actin gene was measured for normalization.

### Immunoprecipitation (IP) and Western Blotting

The NKAPL-152T and NKAPL-152N expression vectors were transfected into HEK 293T cells respectively. And 48 h later, proteins from cell lysates were immunoprecipitated using the phosphothreonine antibody. For immunoprecipitation, the cells were dissolved in a lysis buffer [25 mM HEPES (pH 7.5), 150 mM NaCl, 0.1% Triton X-100, 10 mM MgCl_2,_ and 1 mM EDTA] supplemented with protease inhibitor mixture and phosphatase inhibitor mixture (Roche). The threonine phosphorylated NKAPL proteins were immunoprecipitated with phosphothreonine antibodies (MilliporeSigma, AB1607) conjugated to Dynabeads Protein G (Thermo Fisher Scientific, 10003D, United States). The immunoprecipitated proteins were electrophoresed on NuPAGE 10% BT Gel (Invitrogen) and transferred to nitrocellulose membranes, which were incubated with primary antibody (anti-HA, Cell Signaling Technology, 2367S) and secondary antibody. IRDye and HRP-related signals were respectively detected and quantified using an infrared image analyzer (United States, LI-COR Bioscience) and a luminescent image analyzer (China, Tanon).

### In Utero Electroporation

First, we constructed the *Nkapl* ^*fl/fl*^ mice. The exon1 of Nkapl was flanked with two loxP sequences. A Neomycin resistance cassette (neo) was used for positive selection. A DTA cassette was used for negative selection in embryonic stem (ES) cells. Pregnant *Nkapl* ^*fl/fl*^ mice were deeply anesthetized, and the intrauterine embryos were surgically manipulated as described previously (Tabata and Nakajima, 2001). In brief, pcAGGS carrying Cre and internal ribozyme entry site (IRES) driving enhanced green fluorescent protein (EGFP) was purified without endotoxin. The concentration of the plasmid was adjusted to 2 mg/ml. The plasmid containing 0.02% Fast Green solution was injected into the lateral ventricle of embryos at embryonic day 14.5 (E14.5). The plasmids were delivered into the ventricular zone (VZ) surface of the cortical plate (CP) in the somatosensory cortical region by electronic pulses (Nepa Gene, Japan). The operated embryos were allowed to live within the uterine horn until the time of observation.

### Statistical Analysis

Chi-square tests were used for gender distribution comparison. Differences in continuous variables (including demographic variables, PANSS score, and cognitive performance) were examined using the independent sample t-test. The above tests were first conducted in the whole sample to figure out the differences between patients with EOS and AOS. Considering the small number of participants with AA genotype, we divided the whole into two genotype groups, that is, A Carriers Group including patients with CA and AA genotype, and the CC group including patients with CC genotype. Hardy-Weinberg equilibrium between expected and observed genotype distributions was tested using the χ2 test. Chi-square tests and independent sample t-tests were then used to find the difference between two genotype groups in patients with EOS and AOS, respectively. All the statistical analyses were carried out using the SPSS 26.0 software (SPSS Inc., Chicago, IL, United States). Results were considered significant at a two-tailed *p* < 0.05.

## Results

### Demographic and Clinical Symptoms of the Study Population

A total of 430 patients with schizophrenia were enrolled in the study, including 222 males and 208 females, with an average age of 21.05 years and an average disease duration of 2.44 years. There was no significant difference between the observed value and expected value of genotype frequency for SNP rs1635 polymorphism (*p* > 0.05 in the Hardy-Weinberg equilibrium test), which suggested that the participants were collected randomly from the general population. There were no significant differences in gender distribution, age, age at onset, duration, and PANSS total score between the two genotype groups in patients with EOS and AOS, respectively (Table 1).

**TABLE 1:** Demographic and clinical characteristics of patients with early-onset schizophrenia in two groups of NKAPL rs1635 genotype.

	Early-onset schizophrenia	CC group	*t/*χ^2^	*P*	Adult-onset schizophrenia		*t/*χ^2^	*P*
	AA/CA group				AA/CA group	CC group		
Gender (M/F)^a^	31/38	33/28	1.089	0.297	109/68	73/50	0.152	0.697
Age (years)	21.09 ± 1.63	21.38 ± 2.48	0.796	0.427	20.92 ± 1.93	21.05 ± 1.95	0.562	0.575
Age at onset (years)	16.01 ± 1.06	15.87 ± 1.40	0.662	0.509	19.87 ± 1.50	19.60 ± 1.46	1.542	0.124
Duration (years)	2.34 ± 1.96	2.09 ± 2.22	0.700	0.485	2.50 ± 2.01	2.60 ± 2.25	0.396	0.692
PANSS score	90.04 ± 14.12	90.33 ± 15.79	0.108	0.914	92.12 ± 17.30	90.72 ± 18.14	0.679	0.498

M, male; F, female; PANSS, positive and negative syndrome scale.

Unless otherwise indicated, data are the Mean ± SD.

a These variables were compared by using χ^2^ tests.

### Association Analysis of rs1635 With Cognitive Function

We examined the differences in cognitive performance of MCCB between two genotype groups in EOS and AOS respectively. For patients with EOS, CC (the CC genotype encodes the protein NKAPL-152T) carriers exhibited higher score in cognitive domain of speed of processing (AA/CA group: 55.07 ± 8.35, CC group: 59.44 ± 10.47, *t* = 2.644, *p* = 0.009, Cohen’d = 0.47). CC carriers in EOS patients mainly performed better in TMT (*t* = 2.221, *p* = 0.028) and Fluency Test (*t* = 2.578, *p* = 0.011). There were no significant differences between groups in other cognitive domains. For patients with AOS, we didn’t observe significant performances in all cognitive domains (Table 2). There were no significant differences in cognitive performance between EOS and AOS (all *p* > 0.05, Supplementary Table S1). There were no significant differences in cognitive performance between the two groups in healthy individuals (all *p* > 0.05, Supplementary Table S2).

**TABLE 2:** Association analysis of rs1635 with MCCB cognitive tests.

	Early-onset schizophrenia		*t*	*P*	Adult-onset schizophrenia		*t*	*P*
	AA/CA group	CC group			AA/CA group	CC group		
Speed of Processing	55.07 ± 8.35	59.44 ± 10.47	2.644	0.009**	56.60 ± 7.74	57.63 ± 9.58	1.019	0.309
TMT	48.38 ± 8.56	52.15 ± 10.91	2.221	0.028*	49.57 ± 9.08	50.51 ± 9.28	0.875	0.382
Fluency	52.11 ± 9.57	57.08 ± 12.35	2.578	0.011*	53.56 ± 10.03	55.34 ± 11.37	1.424	0.155
BACS_SC	61.51 ± 9.51	62.57 ± 11.85	0.569	0.570	61.99 ± 9.65	61.81 ± 9.54	0.156	0.876
Attention/Vigilance	51.78 ± 8.53	51.90 ± 8.31	0.079	0.937	51.58 ± 7.63	51.06 ± 8.66	0.555	0.579
Working Memory	55.38 ± 10.04	54.46 ± 8.62	0.555	0.580	55.99 ± 9.27	55.87 ± 10.65	0.107	0.914
Verbal Learning	53.40 ± 8.07	54.06 ± 10.83	0.397	0.692	54.52 ± 9.64	54.58 ± 9.88	0.045	0.964
Visual Learning	57.70 ± 4.92	57.05 ± 6.90	0.620	0.536	57.77 ± 6.14	56.82 ± 6.92	1.254	0.211
Reasoning and Problem Solving	48.65 ± 8.90	50.41 ± 8.01	1.177	0.241	49.72 ± 8.63	50.18 ± 8.62	0.456	0.649
Social Cognition	39.04 ± 8.54	40.24 ± 8.29	0.809	0.420	39.52 ± 8.28	39.56 ± 8.49	0.043	0.966
MCCB Total Score	51.59 ± 4.64	52.52 ± 5.64	0.108	0.914	52.24 ± 4.45	52.29 ± 5.45	0.073	0.942

MCCB, MATRICS, consensus cognitive battery; TMT, Trail Making Test: Part A; fluency, Category Fluency: Animal Naming Test; BACS_SC, Brief Assessment of Cognition in Schizophrenia: Symbol Coding Test.

*p* < 0.01, *p < 0.05.

### NKAPL mRNA Level in 152N Carriers Decreased in Peripheral Blood of EOS Patients

**FIGURE 1:**
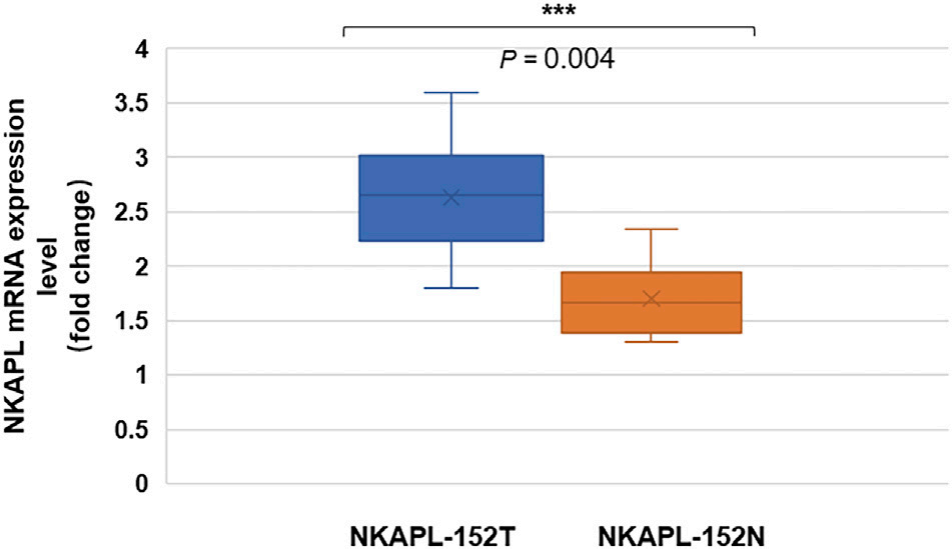
Peripheral blood mRNA expression level of NKAPL-152N was decreased compared with that of NKAPL-152T carriers. The peripheral blood NKAPL mRNA expression level was decreased in 152T carriers (n = 20) than that in 152N carriers (n = 20) in EOS patients, by using the qRT-PCR examination. Data were expressed as mean ± SD, 2-tailed student’s t-test. ***p = 0.004.

We examined the mRNA expression of NKAPL in 40 peripheral blood samples of EOS patients by using quantitative real-time PCR. The fold changes of the NKAPL mRNA level were compared using the t-test between 152T and 152N carriers. The peripheral blood mRNA expression levels of NKAPL in the 152N carriers [n = 20, (2.63 ± 0.82)-fold changes] of EOS patients when compared with that of 152T carriers [*n* = 20, (1.70 ± 0.56)-fold changes] (*p* = 0.004) (Figure 1).

### Expression Quantitative Trait Loci About NKAPL rs1635 in Database.

We attempted to assess the expression quantitative trait loci (eQTL) about rs1635 in the GTEx database. The eQTL for rs1635 in several brain tissues is listed in Table 3, Supplementary figure S1, which was derived from the GTEx database. There is evidence that ZSCAN31 is associated with schizophrenia (Supplementary Figure S2). SNP in ZSCAN31 (rs7759855) had the strongest association with the phenotypes for schizophrenia in the Japanese population (Saito et al., 2014). Methylation in promoter regions of ZSCAN31 in the brain had an effect on schizophrenia (Pineda-Cirera et al., 2022). ZKSCAN3 plays a role in the transcriptional regulation of autophagy (Pan et al., 2017; Barthez et al., 2020; Cho et al., 2021). Autophagy is an important cellular process that maintains homeostasis by recycling damaged organelles and nutrients during cell development and stress. Autophagy dysfunction in the hippocampus, especially in the CA2 region, may relate to deficits of social communication and interaction in schizophrenia patients (Yang and Xu, 2020). Gene expression pattern of ZNF391 in the brain led to the three biotypes, which performed significantly differently in working memory and demonstrated different gray matter volumes in the right inferior frontal orbital gyrus (Barthez et al., 2020). These data provide clues for further exploring the pathogenesis and cognitive impairment mechanism of schizophrenia.

**TABLE 3:** The eQTL analyses for rs1635 gene using the GTEx database.

SNP ID	Gene Symbol	Gencode ID	*p*-value	NES	Tissue
rs1635					
	ZSCAN31	ENSG00000235109.7	0.0000043	0.72	Brain–Frontal Cortex (BA9)
	ZSCAN31	ENSG00000235109.7	0.000055	0.76	Brain–Cortex
	ZSCAN31	ENSG00000235109.7	2.20E-08	1.1	Brain–Cerebellum
	ZSCAN31	ENSG00000235109.7	0.0000022	0.89	Brain–Cerebellar Hemisphere
	ZNF192P1	ENSG00000226314.7	0.00033	0.69	Brain–Cerebellar Hemisphere
	ZKSCAN3	ENSG00000189298.13	0.00039	-0.49	Brain–Cerebellar Hemisphere
	ZNF391	ENSG00000124613.8	0.00017	-0.52	Brain–Cerebellum
	ZSCAN9	ENSG00000137185.11	0.00001	-0.57	Brain–Anterior cingulate cortex (BA24)

### Nkapl Regulates Radial Migration in the Embryonic Cortex

We constructed *Nkapl* ^*fl/fl*^ mice and then investigated Nkapl function in projection neurons during neocortical development. Progenitors in the VZ of *Nkapl* ^*fl/fl*^ embryos were electroporated with the chicken beta-actin (CAG) promoter-driven Cre-IRES-EGFP plasmid to delete Nkapl in late-born (E14.5) neurons. The CAG promoter-driven IRES-EGFP plasmid was used as a control. In Nkapl-deleted embryos, GFP-positive (GFP+) late-born cortical neurons abnormally stayed in the intermediate zone (IZ) and fewer neurons were found in the upper cortical plate (CP) at E17.5 (Figure 3A). The transition from multipolar to bipolar form of the neurons is a key step for neuronal migration and cortical development. *Nkapl* ^*fl/fl:Ctl*^ and *Nkapl* ^*fl/fl:Cre*^ neurons electroporated at E14.5 were analyzed at E17.5 to investigate neuronal morphology in the middle and upper IZ. The proportion of unipolar/bipolar cells was lower in *Nkapl* ^*fl/fl:Cre*^ neurons than in controls (Figure 3B), suggesting that the Multipolar-to-Bipolar transition is affected by Nkapl ablation. The Golgi apparatus marker GRASP65 was labeled to assess the migratory direction of neurons in the IZ. The proportion of *Nkapl* ^*fl/fl:Cre*^ neurons with the Golgi apparatus located toward the CP decreased in the IZ compared with *Nkapl* ^*fl/fl:Ctl*^ neurons (Figure 3C), which is in accordance with a defect in the Multipolar-to-Bipolar transition. These data suggested that Nkapl plays a role in the morphological transition of neurons and migration velocity in late embryos. Neuronal migration is crucial for the development of higher brain functions, including cognitive functions (Gulsuner et al., 2013). Nkapl may affect EOS cognitive function by regulating embryonic cortical development.

### Level of 152N-Phosphorylated NKAPL Is Decreased Compared to 152T-Phosphorylated NKAPL

The rs1635 is a nonsynonymous SNP resulting in a T152N substitution in the NKAPL protein. It is predicted the variant rs1635 prevents the phosphorylation of the nascent NKAPL (Figure 3A), thus we explored the phosphorylation levels of NKAPL-152T and NKAPL-152N. We constructed NKAPL-152T and NKAPL-152N expression vectors and we observed the phosphorylation level of NKAPL-152N was significantly decreased compared to NKAPL-152T (Figure 3B). These data suggest that rs1635 may mediate changes in cognitive function by regulating the phosphorylation level of NKAPL protein.

## Discussion

In this study, we conducted the association analysis of *NKAPL* variant rs1635 with cognitive function in patients with EOS and AOS. The SNP rs1635 was observed to impact cognitive processes of EOS, but not AOS and healthy individuals. Because rs1635 did not affect the cognitive function of healthy individuals, suggesting that it may interact with other genes or environmental factors to affect the cognitive function of EOS. In the cognitive domain for speed of processing, EOS patients with CC genotype performed better on tests of Category Fluency and Trail Making, as well as the composite score for speed of processing, which suggested that rs1635 might play a biological function in the neurodevelopment process that affects information processing in EOS.

According to our results, the SNP rs1635 only affected cognitive processes in patients with EOS and not in AOS, indicating the more contributions of genetics to cognitive impairments of EOS. EOS, defined as the manifestation of psychotic symptoms before 18 years of age (McClellan and Stock, 2013), is a less common and phenotypically more severe form of the disorder, more influenced by genetics and development. Childhood and adolescence are critical periods wherein specific neural systems are undergoing rapid changes such as decreased synaptic density and axon retraction in the prefrontal cortex, which coincide with increased ability in complex high-order cognitive tasks (Vyas et al., 2010). There is a great degree of neural pathology in patients with EOS, with delayed and altered maturation processes in both gray and white matter, and disrupted development of the brain’s normal maturational trajectory (Douaud et al., 2009; Reig et al., 2011). But we didn’t find that rs1635 was associated with cognitive function in healthy individuals. Therefore, the SNP rs1635 located on the *NKAPL* gene may regulate cognitive function with other factors such as genes or environment by affecting cortical development and have an effect during the sensitive period of neurodevelopment.

**FIGURE 2:**
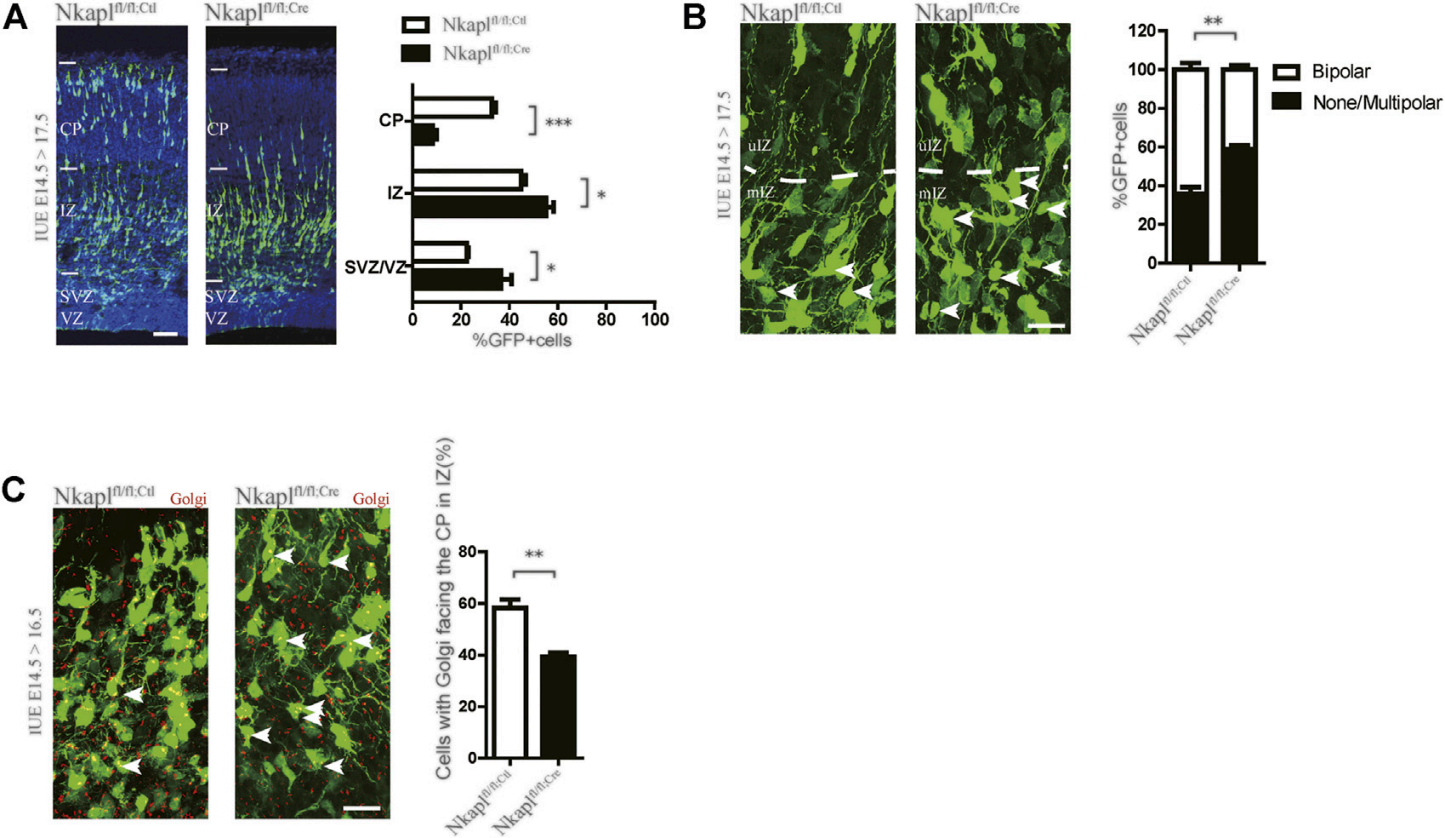
Depletion of Nkapl impairs embryonic radial migration of projection neurons. **(A)** Left panels, *Nkapl* ^*fl/fl*^ brains were electroporated in utero at E14.5 with IRES-EGFP or Cre-IRES-EGFP plasmids and analyzed at E17.5. Nuclei stained with Hoechst to mark distinct CP, IZ, and SVZ/VZ layers. Scale bar, 50 μm. Right panels, the distribution of cortical neurons at E17.5 after electroporation (*n* = 12 slices from three *Nkapl* ^*fl/fl:Ctl*^ mice and *n* = 12 slices from three *Nkapl* ^*fl/fl:Cre*^ mice). **(B)** Left panels, *Nkapl* ^*fl/fl*^ brains were electroporated in utero at E14.5 with IRES-EGFP or Cre-IRES-GFP plasmids and analyzed at E17.5. Arrow, none, or multipolar neurons. Scale bar, 20 μm. Right panels, percentages of unipolar/bipolar and multipolar neurons in each condition (*n* = 12 slices from three *Nkapl* ^*fl/fl:Ctl*^ mice and *n* = 12 slices from three *Nkapl* ^*fl/fl:Cre*^ mice). **(C)** Representative images and quantification of the proportion of neurons with GRASP65 facing the CP in the IZ 2 days after E14.5 electroporation in *Nkapl* ^*fl/fl:Ctl*^ and *Nkapl* ^*fl/fl:Cre*^ cells (*n* = 12 slices from three *Nkapl* ^*fl/fl:Ctl*^ mice and *n* = 12 slices from three *Nkapl* ^*fl/fl:Cre*^ mice). Scale bar, 20 μm. Data were expressed as mean ± SEM, 2-tailed student’s t-test, *p < 0.05; **p < 0.01; ***p < 0.001.

The neurodevelopmental hypothesis is one of the dominant paradigms for schizophrenia and has been widely accepted (McCutcheon et al., 2020). The rs1635 located on the *NKAPL* gene within the extended major histocompatibility complex (MHC) region (6p21.2-p22.3) results in a T152N substitution in the encoded protein and may involve the program for neurodevelopment. The previous study has shown that the *NKAPL* gene affects cortical neuron and synaptic development (Yue et al., 2011). In the ICR mice, RNA interference (RNAi)-mediated knockdown of *NKAPL* expression showed the slow migration of cortical neurons (Yue et al., 2011), which is consistent with our findings (Figure 2A). Our results also found that NKAPL is required for the transition of neurons from multipolar to bipolar (Figure 2B). These results suggest that *NKAPL* may play a role in cortical early developmental processes. In EOS patients, *NKAPL* mRNA level in NKAPL-152N carriers is significantly lower in peripheral blood (Figure 1), Although the level of mRNA in plasma cannot fully represent the level in brain tissue, it still has a certain hinting effect. It is speculated that 152N depletes NKAPL expression and affects embryonic development. As EOS become symptomatic during a critical period for major changes in the neural systems (Paus et al., 2008), and genetic risk factors play a more salient role in EOS (Guo et al., 2021), the regulation of neuronal development by *NKAPL* may be part of the factors affecting cognitive function in early-onset schizophrenia. However, we still need to further explore whether *NKAPL* regulates other functions besides embryonic cortical development and affects the development of cognitive functions.

**FIGURE 3:**
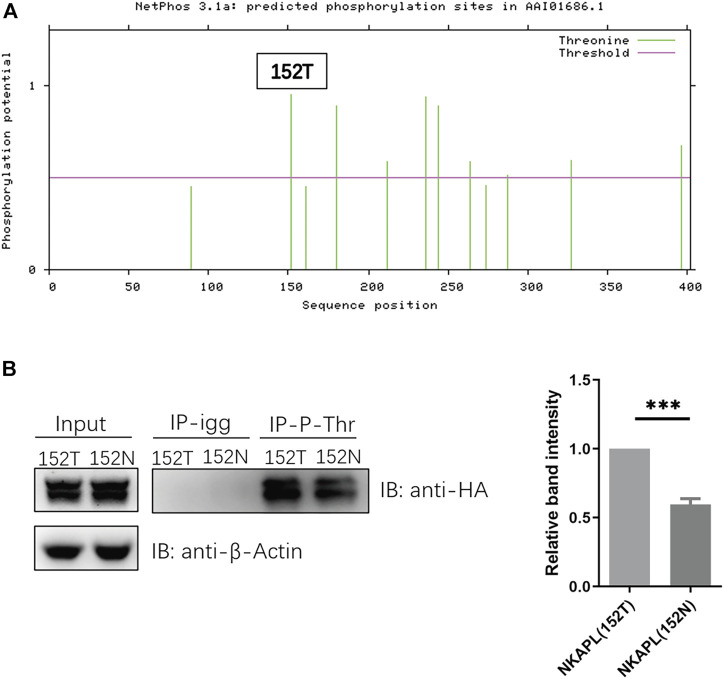
Phosphorylation level of NKAPL-152N is decreased compared to NKAPL-152T. **(A)** Phosphorylation sites prediction using NetPhos 3.1 (https://services.healthtech.dtu.dk/service.php?NetPhos-3.1). **(B)** The protein level of phosphorylated NKAPL is decreased in NKAPL-152N, and a significant decrease was shown by western blotting, *n* = 3 biological replicates in each group. Data were expressed as mean ± SEM, 2-tailed student’s t-test. ***P < 0.001.

Our results demonstrate that rs1635 affects the phosphorylation level of NKAPL protein. Protein phosphorylation of serine, threonine, and tyrosine residues is one of the most prevalent post-translational modifications fundamental in mediating diverse cellular functions in living cells. The results showed phosphorylation level of NKAPL-152N is significantly decreased compared to NKAPL-152T (Figure 3). It is speculated that changes in the phosphorylation level of NKAPL-T152N will affect the expression level of NKAPL protein and neuronal migration during the embryonic stage. But we still need further experiments to verify this conjecture. Numerous studies have shown that phosphorylation levels of different proteins may affect cognitive function. For example, in Alzheimer’s disease, T217-phosphorylation exacerbates wild-type tau hyperphosphorylation with aggravated tau cleavage/fibrillization and cognitive impairments (Wang et al., 2021); in mouse, altering KCC2 phosphorylation resulted in long-term abnormalities in social behavior and memory retention (Moore et al., 2019). We need to further explore the specific biological mechanisms by which NKAPL phosphorylation level mediates cognitive function and whether it mediates neurodevelopmental processes.

There are still several limitations of this study. First, our findings in EOS need to be validated in an independent sample. Second, rs1635 might contribute to cognitive deficits in schizophrenia, but whether it affects cognitive function in healthy individuals remains to be further explored, which might help confirm the genetic effect of rs1635 on cognition. Third, the specific biological mechanism by which it affects cognitive processes is still unclear. Further study on the biological function of SNP rs1635 or *NKAPL* gene may provide a new understanding of the mechanism of cognitive impairment in patients with schizophrenia, thereby providing an attractive treatment method targeting the phosphorylation of NKAPL.

In conclusion, we found that rs1635, located on the *NKAPL* gene, does affect cognitive processes in patients with EOS, but not in patients with AOS. The variant decreases the phosphorylation of NKAPL and decreases its transcription in the peripheral blood, which provides new insights for elucidation of the roles and mechanisms of this risk variant in schizophrenia and helps explore prevention for cognition impairment in EOS.

## Data Availability

The datasets presented in this study can be found in online repositories. The names of the repository/repositories and accession number(s) can be found below: https://doi.org/10.18170/DVN/12DOJZ, 10.18170/DVN/12DOJZ.
