# Supplementary material for: Association of NKAPL rs1635 With Cognitive Function in Early-Onset Schizophrenia
Source: Front Genet. 2022 Jun 21;13:941171. doi: 10.3389/fgene.2022.941171 (PMC9253766; doi:10.3389/fgene.2022.941171)
Supplement: Supplementary file 1 [file DataSheet1.docx]

**Supplementary Materials**

**Table S1 MCCB cognitive tests in patients with early-onset schizophrenia and adult-onset schizophrenia.**

|  | Early-Onset Schizophrenia | Adult-Onset Schizophrenia | *t* | *P* |
| --- | --- | --- | --- | --- |
| Speed of Processing | 57.12±9.62 | 57.02±8.54 | 0.107 | 0.915 |
| TMT | 50.13±9.88 | 49.96±9.16 | 0.177 | 0.860 |
| Fluency | 54.45±11.20 | 54.28±10.61 | 0.144 | 0.886 |
| BACS_SC | 62.01±10.64 | 61.92±9.59 | 0.087 | 0.930 |
| Attention/Vigilance | 51.84±8.40 | 51.37±8.06 | 0.544 | 0.587 |
| Working Memory | 54.95±9.38 | 55.94±9.84 | 0.978 | 0.328 |
| Verbal Learning | 53.71±9.43 | 54.55±9.72 | 0.822 | 0.412 |
| Visual Learning | 57.39±5.92 | 57.38±6.48 | 0.014 | 0.989 |
| Reasoning and Problem Solving | 49.48±8.51 | 49.91±8.62 | 0.477 | 0.634 |
| Social Cognition | 39.61±8.41 | 39.54±8.35 | 0.086 | 0.932 |
| MCCB Total Score | 52.03±5.13 | 52.26±4.88 | 0.452 | 0.652 |

MCCB, MATRICS Consensus Cognitive Battery; TMT, Trail Making Test: Part A; Fluency, Category Fluency: Animal Naming Test; BACS_SC, Brief Assessment of Cognition in Schizophrenia: Symbol Coding Test.

**Table S2 MCCB cognitive tests in healthy individuals.**

|  | Healthy individuals | | | |
| --- | --- | --- | --- | --- |
|  | AA/CA Group | CC Group | *t*/χ^2^ | *P* |
| Gender(M/F) | 127/129 | 125/134 | 0.093 | 0.760 |
| Age(years) | 24.47±3.95 | 24.50±3.70 | 0.087 | 0.931 |
| Speed of Processing | 56.88±8.54 | 57.55±8.63 | 0.879 | 0.380 |
| TMT | 49.58±9.52 | 50.06±9.64 | 0.577 | 0.564 |
| Fluency | 54.26±10.53 | 54.94±10.58 | 0.727 | 0.467 |
| BACS_SC | 62.10±9.43 | 62.35±10.04 | 0.286 | 0.775 |
| Attention/Vigilance | 51.38±7.64 | 51.83±8.17 | 0.646 | 0.519 |
| Working Memory | 56.52±9.83 | 56.30±9.51 | 0.256 | 0.798 |
| Verbal Learning | 54.27±9.34 | 54.81±9.78 | 0.642 | 0.521 |
| Visual Learning | 58.05±6.34 | 57.42±5.89 | 0244 | 0.539 |
| Reasoning and Problem Solving | 50.46±8.98 | 50.60±8.65 | 0.177 | 0.860 |
| Social Cognition | 40.22±8.18 | 38.82±8.56 | 1.895 | 0.059 |

MCCB, MATRICS Consensus Cognitive Battery; TMT, Trail Making Test: Part A; Fluency, Category Fluency: Animal Naming Test; BACS_SC, Brief Assessment of Cognition in Schizophrenia: Symbol Coding Test.

**Supplementary Figure 1**

**According to the Braineac database, the NKAPL rs1635 indicated significant eQTL effects in the following human brain tissues: cerebellar hemisphere, frontal cortex, anterior cingulate cortex, etc.**

**
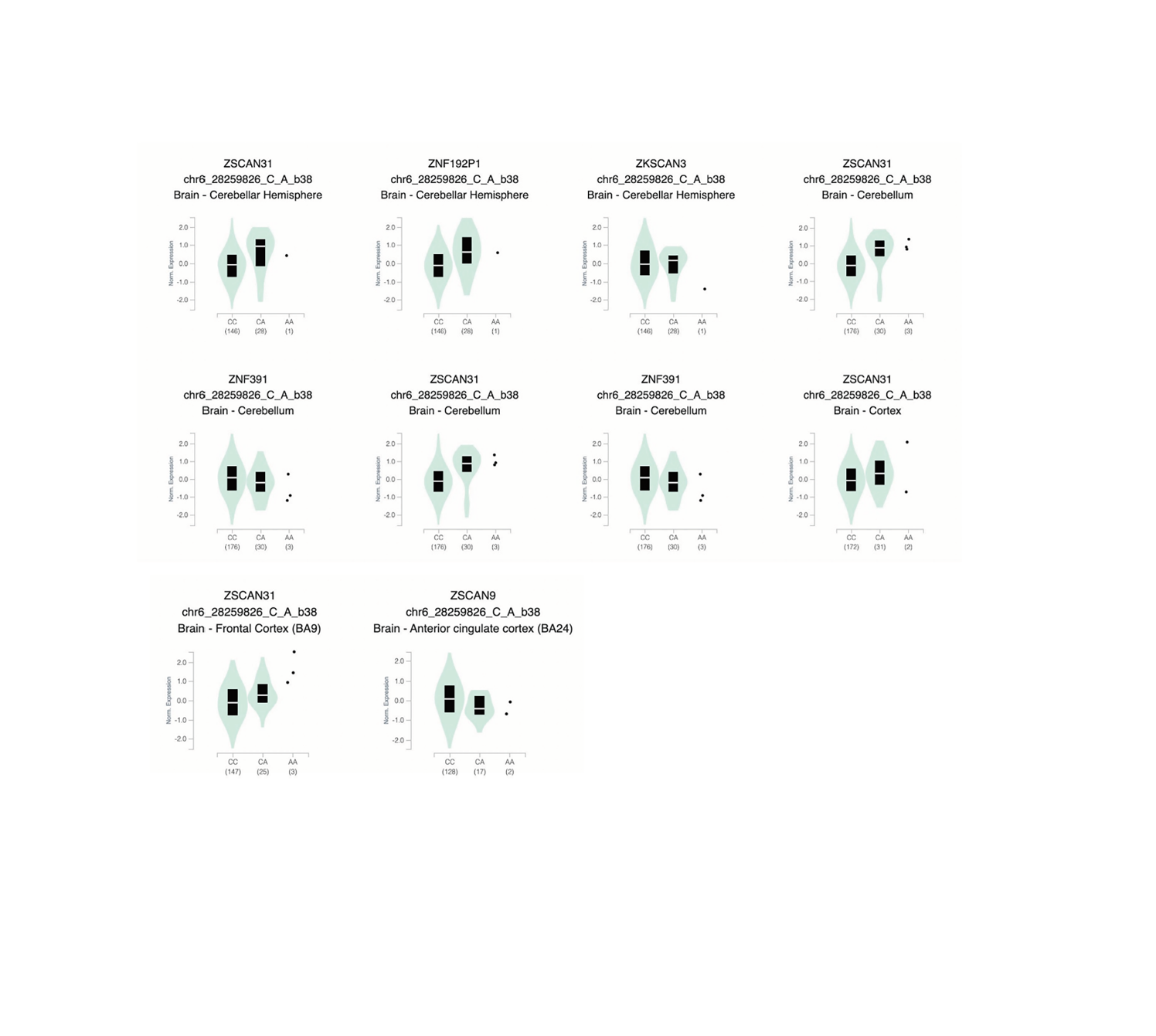
**

**Supplementary Figure 2**

**According to the HBT database, the NKAPL mRNA expresses highly in the cerebellum, frontal cortex, anterior cingulate cortex, etc.**

**
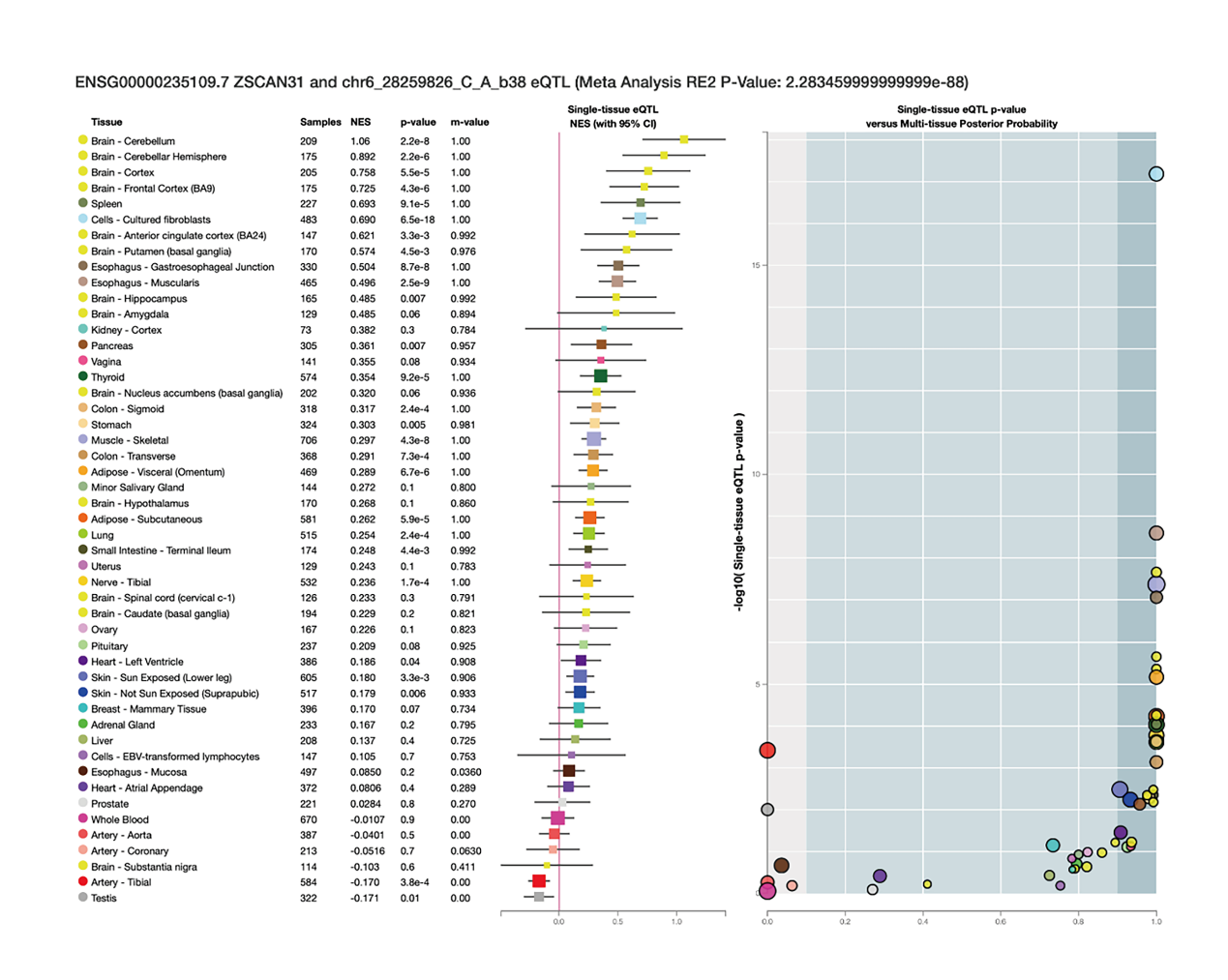
**
